# Genetic Contributions to The Association Between Adult Height and Head and Neck Cancer: A Mendelian Randomization Analysis

**DOI:** 10.1038/s41598-018-22626-w

**Published:** 2018-03-14

**Authors:** Roberta Pastorino, Anna Puggina, Robert Carreras-Torres, Pagona Lagiou, Ivana Holcátová, Lorenzo Richiardi, Kristina Kjaerheim, Antonio Agudo, Xavier Castellsagué, Tatiana V. Macfarlane, Luigi Barzan, Cristina Canova, Nalin S. Thakker, David I. Conway, Ariana Znaor, Claire M. Healy, Wolfgang Ahrens, David Zaridze, Neonilia Szeszenia-Dabrowska, Jolanta Lissowska, Eleonora Fabianova, Ioan Nicolae Mates, Vladimir Bencko, Lenka Foretova, Vladimir Janout, Paul Brennan, Valérie Gaborieau, James D. McKay, Stefania Boccia

**Affiliations:** 1 Section of Hygiene - Institute of Public Health, Università Cattolica del Sacro Cuore, L.go F. Vito, 1, 00168 Rome, Italy; 2 International Agency for Research on Cancer (IARC), Lyon, France; 3 Department of Hygiene, Epidemiology and Medical Statistics, University of Athens School of Medicine, Athens, Greece; 4 Institute of Hygiene and Epidemiology, 1st Faculty of Medicine, Charles University, Prague, Czech Republic; 5 University of Turin, Department of Medical Sciences, Unit of Cancer Epidemiology, Turin, Italy; 6 Cancer Registry of Norway, Oslo, Norway; 7 Cancer Epidemiology Research Program, Catalan Institute of Oncology (ICO), IDIBELL, L’Hospitalet de Llobregat, Catalonia, Spain; 8 CIBER Epidemiología y Salud Pública (CIBERESP), Barcelona, Spain; 9 School of Medicine and Dentistry, University of Aberdeen, Aberdeen, United Kingdom; 10 General Hospital of Pordenone, Pordenone, Italy; 11 Department of Environmental Medicine and Public Health, University of Padova, Padova, Italy; 12 MRC-HPA Centre for Environment and Health, Respiratory Epidemiology and Public Health, National Heart and Lung Institute, Imperial College, London, United Kingdom; 13 University of Manchester, School of Dentistry, Manchester, United Kingdom; 14 University of Glasgow Dental School, Glasgow, Scotland United Kingdom; 15 Croatian National Cancer Registry, Croatian National Institute of Public Health, Zagreb, Croatia; 16 Trinity College School of Dental Science, Dublin, Ireland; 17 Leibniz Institute for Prevention Research and Epidemiology—BIPS, Bremen, Germany; 18 Faculty of Mathematics and Computer Science, University of Bremen, Bremen, Germany; 19 Institute of Carcinogenesis, Cancer Research Centre, Moscow, Russian Federation; 20 Department of Epidemiology, Institute of Occupational Medicine, Lodz, Poland; 21 Department of Cancer Epidemiology and Prevention, M. Sklodowska-Curie Memorial Cancer Center and Institute of Oncology, Warsaw, Poland; 22 Regional Authority of Public Health, Banska Bystrica, Slovakia; 23 Saint Mary General and Esophageal Surgery Clinic, Carol Davila University of Medicine and Pharmacy, Bucharest, Romania; 24 Department of Cancer Epidemiology and Genetics, Masaryk Memorial Cancer Institute and Masaryk University, Brno, Czech Republic; 25 Palacky University, Olomouc, Czech Republic; 26 Section of Hygiene, Institute of Public Health, Università Cattolica del Sacro Cuore, IRCCS Fondazione Policlinico ‘Agostino Gemelli’, Rome, Italy

## Abstract

With the aim to dissect the effect of adult height on head and neck cancer (HNC), we use the Mendelian randomization (MR) approach to test the association between genetic instruments for height and the risk of HNC. 599 single nucleotide polymorphisms (SNPs) were identified as genetic instruments for height, accounting for 16% of the phenotypic variation. Genetic data concerning HNC cases and controls were obtained from a genome-wide association study. Summary statistics for genetic association were used in complementary MR approaches: the weighted genetic risk score (GRS) and the inverse-variance weighted (IVW). MR-Egger regression was used for sensitivity analysis and pleiotropy evaluation. From the GRS analysis, one standard deviation (SD) higher height (6.9 cm; due to genetic predisposition across 599 SNPs) raised the risk for HNC (Odds ratio (OR), 1.14; 95% Confidence Interval (95%CI), 0.99–1.32). The association analyses with potential confounders revealed that the GRS was associated with tobacco smoking (OR = 0.80, 95% CI (0.69–0.93)). MR-Egger regression did not provide evidence of overall directional pleiotropy. Our study indicates that height is potentially associated with HNC risk. However, the reported risk could be underestimated since, at the genetic level, height emerged to be inversely associated with smoking.

## Introduction

Head and neck cancer (HNC) is the seventh most common cancer worldwide, with more than a half million new cases in 2012^1^.

This includes carcinomas of the upper aerodigestive tract (UADT: oral cavity, nasopharynx, oropharynx, hypopharynx, and larynx), the paranasal sinuses, and the salivary glands, being squamous cell carcinoma the most common histopathological type^2^.

Tobacco smoking and alcohol consumption are the main environmental risk factors for HNC^3–5^, although other factors, including infection from human papillomavirus (HPV), low physical activity, poor diet, and low socioeconomic status, affect the risk^6–10^. Adult height has also been observed as a risk factor for HNC^11^, among other cancer outcomes^12–21^. In the prospective NIH-AARP cohort study, with 218,854 participants aged between 50 and 71, it was observed a 34% risk increase for HNC among individuals in the fourth quartile of height^11^. However, inverse associations have also been reported^22^. These inconsistencies could be due to different study designs and potential residual confounding.

To circumvent these limitations of observational studies, Mendelian randomization (MR) is a technique aimed at validation of causal effects using genetics^23^. MR analysis uses an instrumental variable (IV) (e.g., genetic variant that proxies for directly measured exposures) to make causal inferences about the relationship between a risk factor and an outcome. The advantage of using germ-line genetic instruments lays in the fact that they have less probability to be associated with environmental confounders or reverse causation. Additionally, the use of multiple genetic variants as instruments can improve the precision of IV estimates and the statistical power of the study^24,25^. Two strategies exist to combine information on multiple uncorrelated IVs into a single causal estimate: using individual-level genetic data or summary statistics for genetic association. In the former, a polygenetic risk score for the exposure can be tested for risk on case-control samples; in the latter, individual IV casual estimates are combined in an inverse-variance weighted (IVW) fixed-effect meta-analysis^26^. However, an enlarged set of genetic variants is more likely to contain invalid IVs, due to the inclusion of pleiotropic variants which can lead to biased causal effect estimates. In order to overcome this potential issue, MR approaches for data with multiple potentially invalid instruments have been developed. The presence of overall directional pleiotropy on the estimated casual effect can be assessed using an adaption of the Egger regression (MR-Egger)^27^.

Adult height is indeed determined by a combination of genetic factors and environmental exposures, both in utero and during childhood and adolescence. In Caucasian population, heritability of adult height is estimated to account for ~80%, while the remaining ~20% is due to environmental factors^28,29^. Recent genome-wide association studies (GWAS) have been identified hundreds of genomic loci linked to human height^30,31^, which account for approximately 16% of phenotypic variation.

The aim of this MR study is to dissect the causal effect of height on HNC cancer in subjects of European ancestry using height-related genetic variants as proxies for height on HNC cancer samples.

## Methods

### Selection of instrumental variables and study sample

A total of 697 single nucleotide polymorphisms (SNPs) were identified as genome-wide associated (p-value < 5 × 10^−8^), from a recently published GWAS on adult height including 253,288 individuals of European ancestry^31^.

Individual-level genetic data concerning HNC cases and controls were obtained from a recent GWAS on UADT cancers. The GWAS was carried out in 2,091 UADT cancer cases and 3,513 controls from two large European hospital-based multi-center studies. These studies were the International Agency for Research on Cancer (IARC) central Europe (CE) study conducted from 2000 to 2002, in 6 centers from 5 countries; and Alcohol-Related Cancers and Genetic susceptibility in Europe (ARCAGE) study conducted by IARC from 2002 to 2005, in 12 centers from 9 European countries^32^. Cases and controls were matched by age and center with a control/case ratio of 1 for ARGACE and 2–3 for CE study. We conducted quality control steps on these data using PLINK software^33^. Genetic variants and individuals with genotype call rate of less than 95% were excluded for the analyses. We also conducted further exclusions where the genotype distribution clearly deviated from that expected by Hardy-Weinberg Equilibrium (HWE) among controls (genome-wide P threshold of 1 × 10^−7^). Genetic principal components (PCs) for population stratification were estimated using 12,898 genetic variants in low linkage disequilibrium (LD) (R^2^ < 0.01). Genotype imputation has been performed using MACH 1.0^34,35^, and the 1000 Genomes Project ALL panel (Phase I integrated Release 3) as haplotype reference panel. Imputed SNPs were restricted on the basis of imputed accuracy, and only SNPs with higher imputation quality (R^2^) than 0.7 were selected for our analyses.

Available phenotypic data for these samples comprised: age, sex (female vs male), country of origin, height, tobacco smoking (coded as never vs ever smokers), alcohol consumption (coded as never vs ever drinkers) and HNC status.

### Observed risk and power assessment of Mendelian randomization analyses

Initially, we evaluated which was the observed risk for the phenotypic height-HNC status association. HNC status was regressed on standardized height controlling for age, sex, country of origin, tobacco smoking and alcohol consumption status (HNC status ~ height + age + sex + country of origin + smoking status + alcohol consumption status). Then, to evaluate the power to validate the observed risk estimates using the MR approach, power calculations were performed based on the number of total cases and controls and the explained proportion of phenotype variance explained by the set of genetic instruments (16%^29^)^36^.

### Mendelian randomization analyses

Complementary MR approaches were performed in this study. First, a weighted genetic risk score (GRS) of previously selected SNPs was used as IV for adult height. For each genotype, participants received a score of 0, 1, or 2, when carrying 0, 1, or 2 height-increasing alleles, respectively. Each allele dosage was weighted by the per-allele change in 1 standard deviation (SD) of height (6.9 cm) reported in the original published study^31^ (GRS = Σβ_GPi_*IV_i_dosage; where i ranges from 1 to total number of IVs). The derived weighted GRS was tested under an additive model as IV to assess the effect of height on HNC. Firstly, we predicted height from the weighted GRS using a linear model and adjusting by age, sex and 15 PCs (Height ~ GRS + age + sex + 15 PCs). Similarly, we assessed the relationship between the weighted GRS and each measured potential confounding factor, namely age, sex, country of origin, tobacco smoking and alcohol consumption status (e.g., smoking status ~ GRS + age + sex + 15 PCs). Finally, we regressed the disease status on the weighted GRS and adjusted for covariates, including age, sex, country of origin, tobacco smoking, alcohol consumption, and 15 PCs (HNC status ~ GRS + age + sex + country of origin + smoking status + alcohol consumption status + 15 PCs). The obtained coefficient was then evaluated as the estimated effect of adult height on HNC. Additionally, we also examined the height-HNC association separately for men and women, and among HNC studies (CE and ARCAGE).

The other MR approaches were performed using the summary statistics for genetic association of selected SNPs on height (β_GPi_; from height GWAS) and on HNC status (β_GDi_: from HNC status ~IV_i_ + age + sex + country of origin + smoking status + alcohol consumption status + 15 PCs). A causal effect estimate of height on HNC was obtained through IVW fixed-effect meta-analysis of SNP Wald ratios (β_GDi_/β_GPi_), whose weights are described as β_GPi_^2^/Seβ_GD_ (Se: standard error), constraining the regression intercept to zero^36^. If all genetic variants satisfy the IV assumptions, then the IVW estimate is a consistent estimate of the causal effect, as it is a weighted mean of the individual ratio estimates. As a sensitivity analysis of the effect of potential directional pleiotropy on the estimated causal effects, an adaption of Egger regression (MR-Egger) was performed on the Wald ratios without constraining the intercept of the regression^27^. The estimated value of the intercept in the Egger regression is interpreted as an estimate of the average pleiotropic effect across the genetic variants. An intercept term that differs from zero is indicative of overall directional pleiotropy. Similarly, these MR approaches were applied within subgroups of sex and studies. Statistical analysis was performed using Stata software (StataCorp. 2013. Stata Statistical Software: Release 13. College Station, TX: StataCorp LP).

## Results

A total of 2,082 HNC cases (65.4% from ARCAGE study and 82.8% males) and 3,477 controls (37.6% from ARCAGE study and 73.6% males) passed quality control steps. The mean adult height (in cm) among female and male controls was 161.8 (SD, 6.7) and 173.7 (SD, 7.0), respectively, whereas among HNC cases, the mean height among females was 161.6 (SD, 6.3) and among males was 172.3 (SD, 7.0) (Table S1 for the multi-center description).

Among both females and males, the proportion of ever smokers was about 1.5-fold higher in HNC cases than in controls (65.4% vs. 38.2% and 95.3% vs. 74.8%, respectively) (Table 1). Ever smokers were also more frequent in ARCAGE study (78%) compared with Central Europe study (71%). The proportion of ever drinkers among HNC cases was of 77.9% and 97.7%, respectively in females and in males (Table 1). Ever drinkers did not differ among studies (91% in ARCAGE and 92% in Central Europe).

**Table 1:** Population characteristics of the studied sample, stratified by gender.

	Females (n = 1275)			Males (n = 4284)		
	Controls mean (sd) or n (%)	HNC cases mean (sd) or n (%)	P value	Controls mean (sd) or n (%)	HNC cases mean (sd) or n (%)	P value
**Total**	917 (100%)	358 (100%)		2560 (100%)	1724 (100%)	
**Age (years)**	59.8 (11.7)	60.8 (11.7)	0.18	59.4 (10.3)	58.7 (9.7)	0.03
**Height (cm)**	161.8 (6.7)	161.6 (6.3)		173.7 (7.0)	172.3 (7.0)	
**Smoking status**			<0.001			<0.001
Never smokers	567 (61.8%)	124 (34.6%)		641 (25.1%)	81 (4.7%)	
Ever smokers	350 (38.2%)	234 (65.4%)		1916 (74.8%)	1643 (95.3%)	
Missing	0 (0.0%)	0 (0.0%)		3 (0.1%)	0 (0.0%)	
**Drinking status**			<0.001			<0.001
Never drinkers	222 (24.2%)	78 (21.8%)		119 (4.6%)	38 (2.2%)	
Ever drinkers	694 (75.7%)	279 (77.9%)		2441 (95.4%)	1685 (97.7%)	
Missing	1 (0.1%)	1 (0.3%)		0 (0.0%)	1 (0.1%)	

Notes: HNC: head and neck cancer; SD: standard deviation.

**Figure 1:**
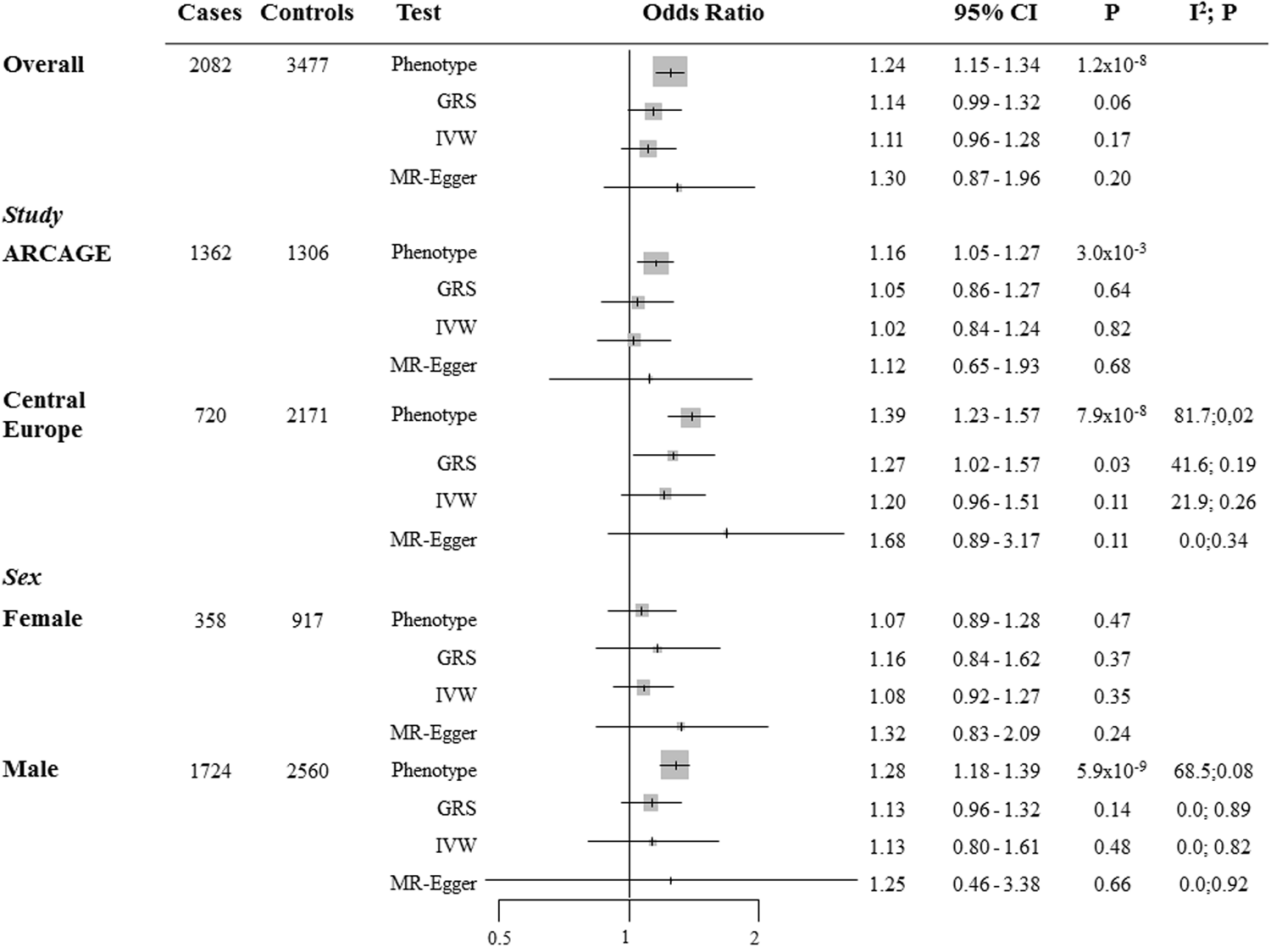
Odds ratios and 95% confidence intervals (95% CI) for the association between the weighted genetic risk score (GRS), the inverse-variance weighted (IVW) estimate, and the Egger Method (MR-Egger) and the risk of head and neck (HNC).

The observed risk of HNC for each SD increase in phenotypic height was of 1.24 (95% CI, 1.15-1.34) (Fig. 1). The analysis stratified by study showed heterogeneity on the risk estimates, each SD increase in height provided an OR = 1.16 (1.05–1.27) for ARCAGE and an OR = 1.39 (1.23–1.57) for central Europe (Fig. 1). Similarly, different risk estimates were observed in the analysis stratified by gender, each SD increase in height provided an OR = 1.28 (1.18–1.39) for men and an OR = 1.07 (0.89–1.28) for women (Fig. 1).

Power estimates of MR analyses to validate observed risk estimates can be observed in Figure S1. The power to validate a risk increase of 1.24 was 81.5%.

Among those SNPs identified as instruments for height, a total number of 599 SNPs resulted with high imputation quality and were genetically independent (linkage disequilibrium R^2^ < 0.01), as listed in Table S2, and were thus used to derive the weighted GRS. None of the 599 SNPs was associated with HNC risk (P < 1 × 10^−5^, Table S2).

The weighted GRS was normally distributed in both HNC cases and controls. The GRS was strongly associated with height (one unit of GRS equaled to 5.05 cm of height, 95% CI (4.65–5.46)). The analysis of the relation between the GRS and potential measured confounders revealed that the GRS was not associated with age (P-value = 0.14), country of origin (P-value = 0.85), and alcohol consumption (P-value = 0.14). However, the GRS was associated with sex (OR = 1.21, 95% CI (1.04–1.41)), and tobacco smoking (OR = 0.80, 95% CI (0.69–0.93)).

Regarding HNC status, each SD increase in height (6.9 cm in Wood *et al*.^29^) provided an OR = 1.14 (95% CI, 0.99–1.32). In the analysis stratified by study, this risk increase was observed for the Central Europe study (OR = 1.27 (1.02–1.57)), but not for ARCAGE (OR = 1.05 (0.86–1.27)) (Fig. 1). Conversely, the risk increase among sex was similar (OR = 1.13 (0.96–1.32) for men and OR = 1.16 (0.84–1.62) for women) (Fig. 1).

The IVW approach provided similar risk estimates for the overall sample (OR = 1.11 (0.96–1.28)) and for the stratified analyses than GRS results (Fig. 1).

Finally, since there was no evidence that the MR-Egger regression intercept was different from zero (data not shown), for both the overall and the stratified analyses, no overall directional pleiotropic effect was detected biasing our previous causal effect estimates. MR-Egger causal estimates can be observed in Fig. 1.

As further sensitivity analyses, we estimated the MR overall effects of height on HNC risk using a set of 448 SNPs with a higher imputation quality (R^2^ > 0.9). The results from GRS analysis did not differ from previous ones (OR pooled sample = 1.16 (0.99–1.37); OR Central Europe study = 1.32 (1.03–1.70); OR ARCAGE = 1.03 (0.83–1.29); OR men = 1.06 (0.72–1.55); and OR women = 1.16 (0.97–1.39)).

## Discussion

The aim of this study was to validate the observed association between height and HNC using genetic proxies for height and providing causal effect estimate free from confounding effects.

Our study indicates that adult height is potentially associated with HNC risk. However, height emerged to be inversely associated with smoking. Therefore, if shorter individuals are more likely to smoke, this could be masking the height-HNC relationship, and the reported effect of height on HNC could be underestimated. Additionally, using complementary MR approaches, the MR risk estimates were in the same risk direction of the conventional phenotypic analysis, but providing lower risk estimates. This could suggest that the observed risk effect of phenotypic height on HNC could be considered a real causal estimate for HNC (since our genetic effects could be underestimated), and/or that the real causal effect is moderate and there exist other factors correlated with phenotypic height that also contribute to HNC risk. The slightly observed study heterogeneity could be partially due to different proportion of ever smokers, being the proportion of smokers higher in the ARCAGE and, thus, attenuating the HNC risk estimate. Additionally, the gender heterogeneity that was found in this study has already been discussed by Walter *et al*., that reported height as an important explanatory factor for the excess risk for men for many shared-site cancers^37^.

A direct biological explanation for the recently observed associations between height and HNC can be hypothesized. The loci found in GWAS are enriched for genes encoding for cytoskeleton and extracellular matrix proteins, proteasis, cell cycle controllers, metabolic enzymes, chromatic molecules, transcription factors and other signaling proteins mainly controlling skeletal growth, body metabolism, cell growth and division regulation, cellular differentiation, senescence and programmed death^38^. Human stature and tumor development appear to share fundamental control mechanisms. Such activities play crucial roles in tumor growth and malignant progression, in support of findings that relate height with cancer risk.

Additionally, the controversy at the basis of the causal relationship between height and HNC risk could be explained through independent effects of environmental risk factors towards both height and HNC risk, or indirect effects of height on HNC risk mediated by behavior risk factors. Examples of the former would be long term effects of in utero nutrition and exposure to hormones, psychological well-being during childhood, the timing of puberty, family social class, and crowded housing which increase the risk of shorter stature in adulthood^15,39^. Examples of the latter would be height determining several aspects of living conditions, with shorter height leading to lower levels of education, lower job status and less income, which was revealed by a MR study, particularly in men^40^. Thus, it appears that adult height could directly increase the risk of HNC, but generate, at the same time, controversial results for HNC risk, through direct or indirect determination of human behavior (our findings that taller stature negatively associates with tobacco smoking, and the heterogeneity observed among studies).

Some limitations can be derived from our study. First, MR assumptions are not completely satisfied. Those include that the genetic IV must be associated with the risk factor of interest, must be independent of potential confounders, and can only affect the outcome through the risk factor. The first assumption is satisfied since IVs were identified from the largest GWAS on height. However, the other two assumptions are not feasible to validate. In our study, we tested the association of our GRS with some potential HNC risk factors, and the GRS was found to be negatively associated with tobacco smoking. This result implies potential bias in our estimate of the effect of height on HNC risk, potentially underestimating the true causal effect. Second, the use of a large number of SNPs as instruments raises the chance to introduce bias due to pleiotropy in our MR results. However, the use of complementary MR approaches with different sensitivity to these pleiotropic effects providing similar risk estimates gave robustness to our results. Finally, since we were not able to stratify our analysis by HNC subtypes, we cannot provide a measure of the association between height and the specific HNC subtypes.

In conclusion, our MR study reported an inverse association between adult height and tobacco smoking and a potential association between adult height and HNC. Given the quite large disparities in population average height, even within Europe, and the quite different trends in human adult height^40,41^, our MR study could have potential implications for public health interventions.

## Electronic supplementary material

Supplementary Information
